# Methylation changes induced by a glycodendropeptide immunotherapy and associated to tolerance in mice

**DOI:** 10.3389/fimmu.2022.1094172

**Published:** 2022-12-14

**Authors:** Rafael Núñez, María J. Rodríguez, Clara Lebrón-Martín, María del Carmen Martín-Astorga, Francisca Palomares, Javier Ramos-Soriano, Javier Rojo, María J. Torres, José Antonio Cañas, Cristobalina Mayorga

**Affiliations:** ^1^ Laboratory of Allergy, Allergy Research Group, Instituto de Investigación Biomédica de Málaga-Plataforma Andalusian Centre for Nanomedicine and Biotechnology (IBIMA-BIONAND), Málaga, Spain; ^2^ Department of Medicine, Universidad de Málaga (UMA), Málaga, Spain; ^3^ Laboratory of Glycosystems, Institute of Chemical Research (IIQ), Spanish National Research Council (CSIC)- Universidad de Sevilla, Sevilla, Spain; ^4^ Clinical Unit of Allergy, Hospital Regional Universitario de Málaga, Málaga, Spain

**Keywords:** dendritic cells, epigenetic changes, immunomodulation, lipid transfer proteins, sublingual immunotherapy

## Abstract

**Introduction:**

Allergen-specific immunotherapy (AIT) is applied as treatment to rise tolerance in patients with food allergies. Although AIT is thoroughly used, the underlying epigenetic events related to tolerant induction are still unknown. Thus, we aim to investigate epigenetic changes that could be related to tolerance in dendritic cells (DCs) from anaphylactic mice to lipid transfer proteins, Pru p 3, in the context of a sublingual immunotherapy (SLIT) with a glycodendropeptide (D1ManPrup3) that has demonstrated tolerant or desensitization responses depending on the treatment dose.

**Methods:**

Changes in DNA methylation in CpG context were determined comparing Sensitized (Antigen-only) animals and two groups receiving SLIT with the D1ManPrup3 nanostructure (D1ManPrup3-SLIT): Tolerant (2nM D1ManPrup3) and Desensitized (5nM D1ManPrup3), against anaphylactic animals. DNA from lymph nodes-DCs were isolated and then, Whole Genome Bisulphite Sequencing was performed to analyze methylation.

**Results:**

Most differentially methylated regions were found on the area of influence of gene promoters (DMPRs). Compared to the Anaphylactic group, the highest value was found in Desensitized mice (n = 7,713 DMPRs), followed by Tolerant (n = 4,091 DMPRs) and Sensitized (n = 3,931 DMPRs) mice. Moreover, many of these epigenetic changes were found in genes involved in immune and tolerance responses (Il1b, Il12b, Il1a, Ifng, and Tnf) as shown by functional enrichment (DCs regulation, B cell-mediated immunity, and effector mechanisms).

**Discussion:**

In conclusion, different doses of D1ManPrup3-SLIT induce different DNA methylation changes, which are reflected in the induction of distinct responses, tolerance, or desensitization.

## 1 Introduction

Over recent years, food allergy (FA) has experienced a relevant increase worldwide and constitutes a health and socio-economic burden (1). In most cases, the avoidance of the food allergen is the unique treatment, which does not prevent the risk of suffering an allergic reaction (2). Thus, developing effective and safe treatments for FA is crucial; however, only allergen-specific immunotherapy (AIT) complies these two requirements currently (3).

FA to non-specific lipid transfer protein (nsLTP), especially to Pru p 3, the major peach allergen, can lead to severe reactions and cross-reactivity with other nsLTPs from numerous foods and pollens, producing a complex clinical pattern: LTP syndrome (4). Food avoidance, as treatment for LTP-allergic patients, is deeply convoluted due to the multi-taxonomic allergenic sources that can trigger an allergic reaction (5). Despite the concerns about its long-term effectivity (6), AIT has shown to reduce LTP allergy (7). Therefore, the study of biomarkers as predicting tools to monitor the treatment response is of great relevance. Our research group has demonstrated that sublingual immunotherapy (SLIT) for LTP-allergic individuals is clinically effective. This SLIT turns the sensitization profile from a Th2 into a Th1/T regulatory (Treg) pattern, increasing allergen-specific IgG_4_ and IL10^+^ Treg cells, while decreasing allergen-specific IgE (sIgE) and Th2/Th9 effector cells (8, 9). Furthermore, in a Pru p 3 anaphylactic mouse model, SLIT with Pru p 3 conjugated to an adjuvant mannose dendron to generate the corresponding D1ManPrup3 glycodendropeptide (GDPs) showed protection from Pru p 3-induced anaphylaxis, diminishing Th2 response enhancing Th1/Treg cells (10). Remarkably, in this study was also observed a SLIT dose-dependent effect, with the higher dose (5 nM D1ManPrup3) inducing temporary desensitization, whereas the lower dose (2 nM D1ManPrup3) producing long-lasting tolerance (10). Moreover, our group demonstrated that tolerance-inducing effects of SLIT with Pru p 3 were orchestrated by dendritic cells (DCs), and showed that the stimulation with this nanostructure induces important changes in DCs surface markers in allergic patients compared to tolerant subjects (11). Additionally, a plethora of studies have described that DCs are major players in immunomodulation during AIT (9, 11, 12).

Taking all previous results into account, our research group studied, in the same anaphylactic mouse model treated with D1ManPrup3-SLIT, the changes on gene expression in DCs (one of the key players of immunomodulation) that could discriminate desensitized animals from long-term tolerant animals (13). An initial differential gene expression study in mice showed that Pru p 3-induced anaphylaxis alters DCs genes involved signal detection (including *Cd14*), Th2 responses, mast cell activation, as well as diverse cytokines and cell recruitment (14). Furthermore, the evaluation of gene expression changes between desensitization and tolerance showed that several differentially expressed genes (DEGs) pointed to DCs tolerogenic behavior in both transcriptional profiles *via* mTOR activation after TLR4 stimulation; however, the vast majority of the leading DEGs involved were specific to desensitized or tolerant animals DCs (13). Such specificity, at transcriptional regulation level, involved different underlying tolerogenic mechanisms and/or time course. Therefore, epigenetic changes could be preserved for a longer period, allowing the study feasibility.

Epigenetic regulation, to which DNA methylation belongs, has been linked to FA pathogenesis as another component (15). Likewise, epigenetic regulation has been studied as part of immunomodulation processes of the same nature as AIT (16). Given that expression studies at RNA or protein level could reduce the time-period in which differences can be detected, our main objective is to study epigenetic changes in DCs, as DNA methylation changes emerging after SLIT could last longer. These epigenetic changes may not only persist over time, but also be transmitted to offspring, with the tolerance responses persisting over the time and alongside generations, as other studies have shown in an allergic mouse model (17). In addition, the presence of epigenetic changes in promoter regions of several key genes in the development of tolerance and allergic responses may give rise to a gene expression signature that could be used as a predictor of tolerance responses, as seen in a previous allergen sensitized asthma mouse model (18). Finally, the present study could help in the search of which are the key genes specific of DCs involved in the tolerance development or in the transitory desensitization, supporting previous works performed in gene expression (19).

The novelty and strengths of our work lie in the use of Whole Genome Bisulphite sequencing (WGBS-seq) to study DNA methylation changes that could either differentiate desensitized and tolerant animals or explain the divergence of the underlying mechanisms of a dose-dependent AIT immunomodulatory treatment, aiming to obtain steady prognostic biomarkers to detect long-term tolerance induction after D1ManPrup3-SLIT.

## 2 Materials and methods

### 2.1 Anaphylactic mouse model: Sensitization, immunotherapy, and challenge

BALB/c female mice of four to five week of age (Janvier Lab, Le Genest-Saint-Isle, France) were used. Experimental animal procedures were performed following the international standards of animal welfare. All protocols were approved by the Animal Experimentation Ethics Committee of Andalusian Centre for Nanomedicine and Biotechnology (BIONAND, Malaga, Spain).

**Figure 1:**
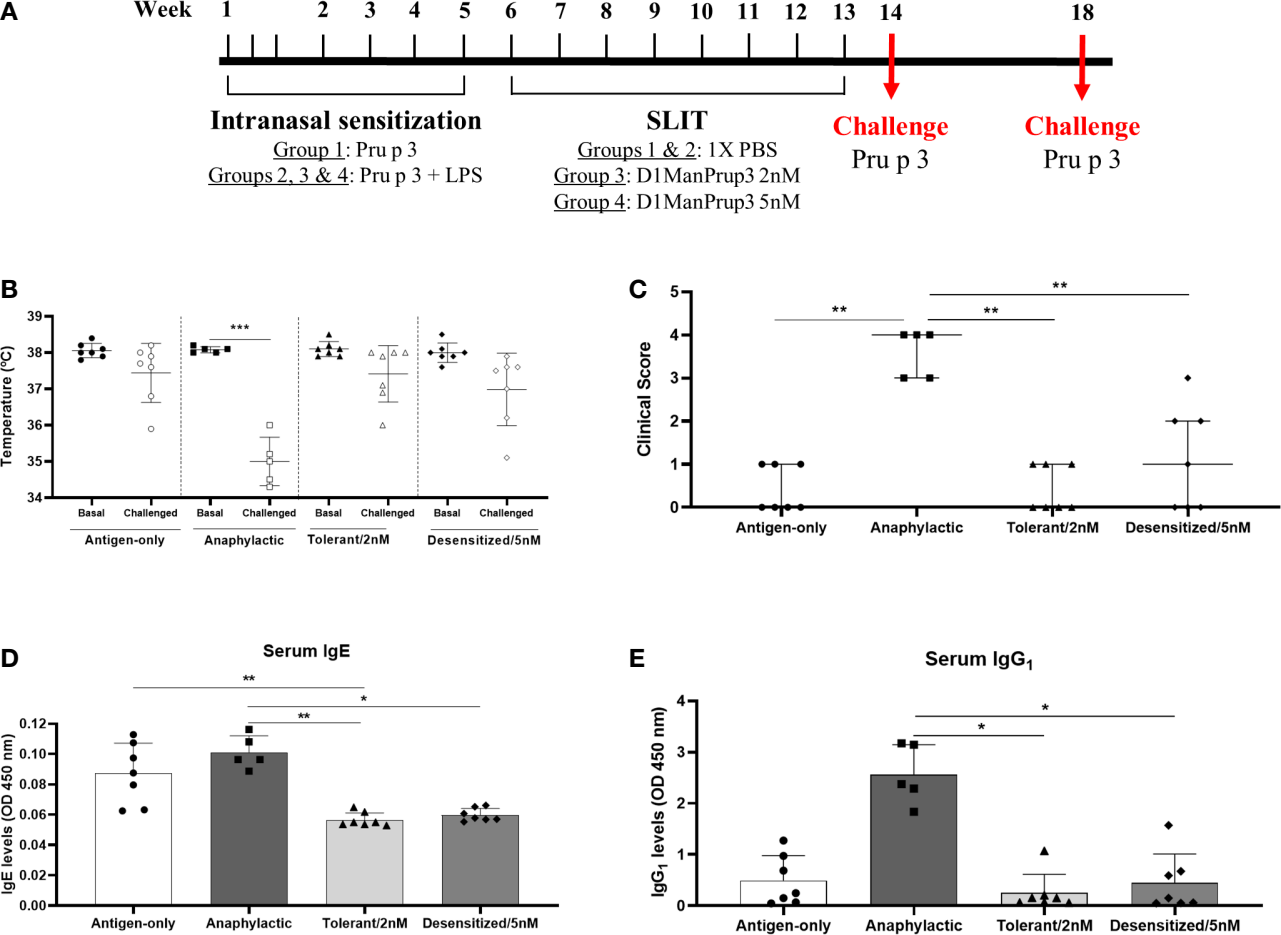
Establishment of anaphylactic mouse model and *in vivo* evaluation of anaphylaxis after SLIT. **(A)** Overview of the experimental design. Mice were sensitized with 20 µg of natural Pru p 3 (Antigen-only) or 20 µg of natural Pru p 3 plus 20ng LPS (the rest of groups), then SLIT was administered during 8 weeks, and challenge was performed one or five weeks after finishing treatment. **(B)** Changes in temperature upon challenge in Antigen only, Anaphylactic, Tolerant/2nM and Desensitized/5nM groups. **(C)** Clinical symptoms upon challenge in Antigen only, Anaphylactic, Tolerant/2nM and Desensitized/5nM groups. **(D)** *In vitro* evaluation of Prup3-sIgE serum levels after challenge. **(E)** *In vitro* determination of Prup3-sIgG1 levels in serum from mice challenged. * *p* < 0.05; ** *p* < 0.01; *** *p* < 0.001.

Animals were divided into four groups: 1) Sensitized, non-anaphylactic (Antigen-only) (n = 7); 2) Anaphylactic, non-treated (Anaphylactic) (n = 7); 3) Anaphylactic, treated with 2 nM D1ManPrup3 (Tolerant) (n = 7); 4) Anaphylactic, treated with 5 nM D1ManPrup3 (Desensitized) (n = 7); as previously described (10) and according to experimental design showed in **Figure 1A**. The procedure used to generate anaphylactic mice was performed using Pru p 3 in combination with lipopolysaccharide (LPS) once a week for five weeks, as described (20). Mice were anaesthetized with isoflurane and then, sensitized intranasally with 20 µg/dose of natural Pru p 3 (Bial Laboratory, Zamudio, Spain) plus 20 ng/dose of LPS (InvivoGen, San Diego, CA, USA) in a final volume of 12µl. Mice from Group 1 (antigen-only) only received Pru p 3 without LPS inducing sensitization but not anaphylactic response.

Preparation of synthetic glycodendropeptide D1ManPrup3 was performed as described previously (21). After the synthesis, this nanostructure was purified by high performance liquid chromatography (HPLC) and characterized by mass spectrometry.

Once the sensitization was completed, at 6-week, D1ManPrup3-SLIT was started following the procedure described (10, 21) and according to experimental design showed in **Figure 1A**. Mice from Groups 3 and 4 were treated once a week for 8 weeks with 2 nM or 5 nM of D1ManPrup3-SLIT, respectively, in a final volume of 10 µl. Mice from Groups 1 and 2 received 10 µl of 1X PBS sublingually. In allprocedures corresponding to treatment, with GDPs or 1X PBS, mice were anaesthetized to assure the compound was maintained sublingually.

One week after completing SLIT, mice were challenged with one intraperitoneal dose of 100 μg of natural Pru p 3 (Bial Laboratory) in a final volume of 100 µl. Anaphylaxis or tolerance were evaluated within 30-40 min after the challenge measuring changes in body temperature with a rectal probe, and assessing physical and behavioral symptoms according to a scoring system (22). Additionally, to assess the long-term response, mice were challenged with one intraperitoneal dose of natural Pru p 3 (100 μg) five weeks after the last doses of SLIT (**Figure 1A**) to confirm tolerance or desensitization.

After the challenge and *in vivo* evaluation, mice were anaesthetized intraperitoneally and bled from the retro-orbital plexus; sera were stored at -20°C for humoral studies. Immediately after bleeding, and while anesthetized, they were sacrificed by cervical dislocation by applying pressure to the neck and dislocating the spinal column from the skull, being performed by trained and authorized personnel. Then, the spleen and lymph nodes (axillary, maxillary, and mesenteric) were removed aseptically and homogeneously through a cell strainer 40 μm (Corning, NY, USA) to obtain a single-cell suspension.

### 2.2 Lymph nodes DCs isolation

DCs from axillary, maxillary, and mesenteric lymph nodes were purified by positive selection using CD11c MicroBeads Ultrapure Mouse kit (Miltenyi Biotec, Bergisch Gladbach, Germany), following manufacturer’s recommendations. The CD11c^+^ cell fraction was maintained in RLT Buffer at -80°C until use.

### 2.3 Whole genome bisulphite sequencing

DNA was extracted from isolated DCs with DNeasy Blood & Tissue Kit (Qiagen, Hilden, Germany) according to manufacturer’s guidelines. DNA quality control, bisulphite conversion, library construction, and sequencing were performed by Novogene (Beijing, China) (23).

### 2.4 Bioinformatic analysis

Sequenced libraries were processed to obtain the methylation ratio of each cytosine in CpG context. First, in the quality control step, low-quality or adapter containing reads were trimmed with Trimmomatic (24). Next, WGBS-seq libraries were aligned against mouse genome (GRCm39) with bwa-meth (25), a BS-seq dedicated aligner based on Burrows-Wheeler Aligner, followed by a deduplication step with Picard (26). Then, cytosines methylation was extracted from each deduplicated library with MethylDackel, excluding likely variant sites.

Following all these processing steps, differential methylation analysis was conducted with MethylKit R package (27), using a sliding window-based algorithm to search for regions overlapping with gene promoters and with significant differences after multiple testing corrections. Regions found with q-value (adjusted p-value) less than 0.05, with an absolute methylation difference bigger than 5% and overlapping with at least one gene promoter were deemed as differentially methylated promoter regions (DMPRs). Also, DMPRs were annotated to their respective genes with R package ChiPseeker (28).

Later, with those annotated genes, a functional enrichment in gene ontology terms and KEGG pathways was conducted aided by R package clusterProfiler (29). Additionally, Venn diagrams, volcano plots and others were constructed with the following R packages, VennDiagram (30), EnhancedVolcano (31) and clusterProfiler (29). **Figure S1** summarizes the applied workflow.

### 2.5 Statistical analyses

Data is expressed as mean ± standard deviation (SD) for parametric data, or median and interquartile range (Q1-Q3) for nonparametric data. Normality was analyzed using the Shapiro-Wilk test. Mann-Whitney test was used to compare paired groups, and Kruskal-Wallis test with Dunn’s *post hoc* test were used to compare multiples groups. Statistical analyses were performed using GraphPad Prism 8 (GraphPad Software Inc., San Diego, CA, USA).

## 3 Results

### 3.1 Different D1ManPrup3-SLIT doses induce tolerant or desensitization in the anaphylactic mouse model

One week after the final dose of SLIT, mice were challenged to confirm the anaphylactic or tolerant responses. After challenge with Pru p 3, only the mice from Anaphylactic group (Group 2) suffered a significant drop in the body temperature (Basal: 38.08 ± 0.08°C vs Challenged: 35.00 ± 0.67°C, *p* < 0.001) (**Figure 1B**), and more severe behavioral and clinical symptoms (**Figure 1C**). Moreover, levels of Pru p 3-sIgE (**Figure 1D**) and -sIgG1 (**Figure 1E**) were decreased in all groups of mice in comparison to Anaphylactic group, showing significant differences in Tolerant/2 nM (sIgE: 0.056 ± 0.005 vs. 0.101 ± 0.011 OD 450 nm, *p* < 0.01; and sIgG_1_: 0.251 ± 0.365 vs. 2.563 ± 0.581 OD 450 nm *p* < 0.05) and Desensitized/5 nM (sIgE: 0.059 ± 0.004 vs. 0.101 ± 0.011 OD 450 nm, *p* < 0.05; and sIgG_1_: 0.451 ± 0.561 vs. 2.563 ± 0.581 OD 450 nm *p* < 0.05) mice in both parameters evaluated. Moreover, it was observed that Pru p 3-sIgE serum levels in Antigen-only group were similar to Anaphylactic group (0.087 ± 0.020 vs. 0.101 ± 0.011 OD 450 nm, *p* > 0.05), in line with our previous results (10), suggesting that the anaphylaxis development may require sIgE but also sIgG_1_. Two mice from Group 2 were excluded from the further study because there was no change in temperature and no evident symptoms.

Tolerant vs desensitization responses were assayed at 5 weeks after the last dose of SLIT, and tolerance was observed in the group of mice treated with 2 nM D1ManPrup3 (Group 3), but not in Group 4 treated with 5 nM D1ManPrup3 (data not shown). These results are in line with our previous findings (10).

### 3.2 WGBS-seq and read alignment

All the 26 mice’s DCs DNA samples (5 for Anaphylactic group and 7 per each other group) that passed the quality control criteria, with bisulphite conversion of > 99%, were sequenced to obtain ~ 300,000,000 reads/sample, of which on average more than 95% aligned to the genome, and more than 80% were retained after deduplication. This resulted in a mean coverage of 20 for each chromosome (more than 100 for mitochondrial) with an 80% of mean methylation on the CpG context (data not shown).

### 3.3 D1ManPrup3-SLIT induces dose-dependent methylation changes

The principal purpose was to examine the epigenomic changes arising from anaphylactic mice after receiving SLIT (Groups 3 and 4). Methylation differences were obtained comparing Antigen-only, Tolerant, and Desensitized mice groups against Anaphylactic group; upmethylated/downmethylated regions exhibit a higher/lower percentage of methylation than the Anaphylactic group, respectively. Besides, the analysis was focused on DMPRs, as they are potentially more prone to influence gene expression.

**Figure 2:**
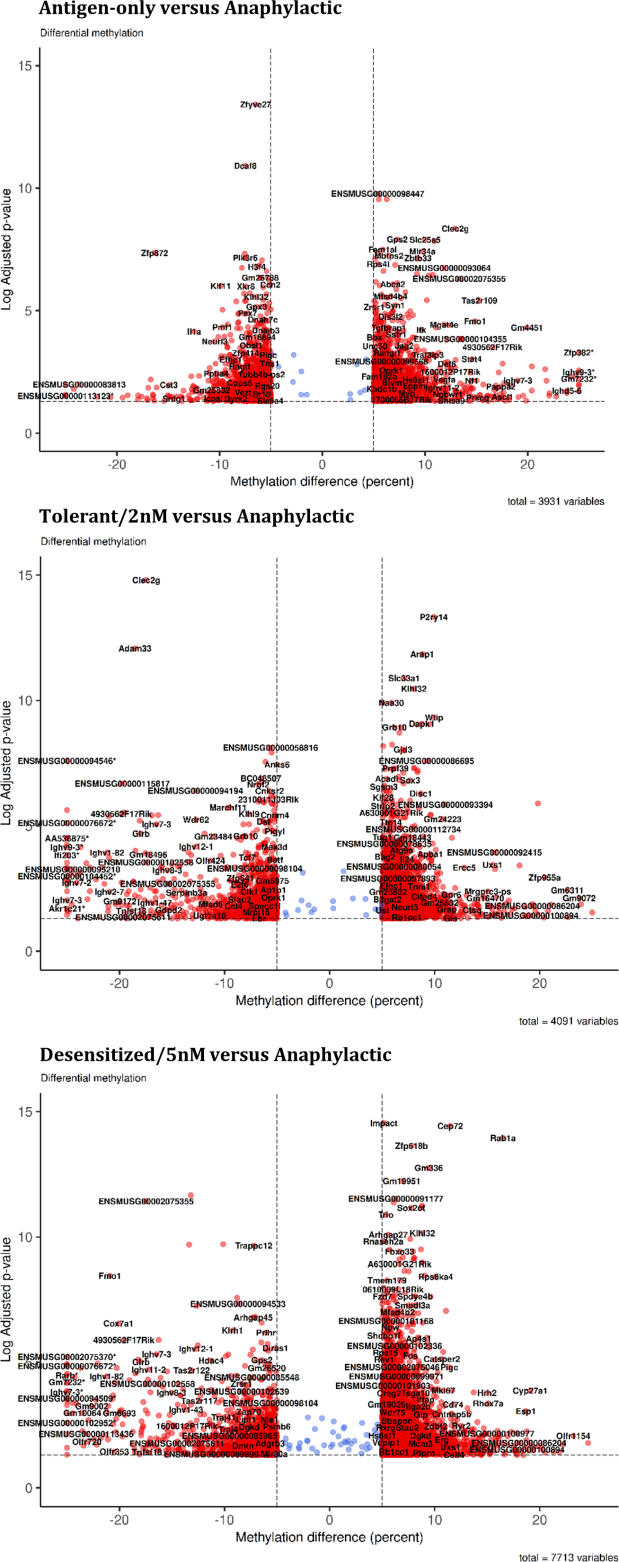
Global changes in promoters cytosines methylation. Volcano plot for each comparison in the summary table. *DMPR with abs (methylation difference) > 30%.

First, the greatest number of DMPRs was found in Desensitized group (7,713), followed by Tolerant (4,091) and Antigen-only (3,931) groups (**Table 1**). Moreover, it was observed that DMPRs number was correlated with the long-term ability to suffer anaphylaxis induced by Pru p 3 (**Table 1**); these differences at quantity level were mostly due to upmethylated DMPRs. Moreover, we found multiple genes with more than one DMPRs affecting its promoter (**Table 1**). Additionally, DMPRs dispersion from the center of the volcano plots (**Figure 2**) corroborated the differences shown in **Table 1**, as methylation changes in Desensitized group were larger and with higher levels of significance. Even though in Tolerant and Antigen-only groups the quantity of DMPRs obtained was similar, the differences in methylation in Tolerant mice were more pronounced both in significance and magnitude. Complete and full details of the differential methylation at promoter regions level found in all comparisons are shown in **Table S1**.

**Table 1:** Number of differentially methylated promoter regions (DMPRs) found in different comparisons against Anaphylactic group.

Differentially Methylated Regions	Antigen-only versus Anaphylaxis	Tolerant/2nM versus Anaphylaxis	Desensitized/5nM versus Anaphylaxis
All	3931	4091	7713
Upmethylated	2068	2726	6181
Downmethylated	1863	1365	1532
Top 50 (by adjusted p-value)	Zswim1, Ybx3, Zfyve27, Dcaf8, Pigo, Yipf6, Clec2g, Gps2, Slc25a5, Fem1al, Mir34a, Zfp872, Pcdh19, Mbtps2, Pik3r6, Mfsd6l, Zbtb33, Fam90a1b, Gltpd2, Rps4l, P4htm, Mn1, H3f4, Scand1, 1700029J07Rik, Ncf4, Tceanc, Klhl24, Gm26788, Dst, Slc1a5, Mir1905, Bmp8b, Slc13a5, Abca2, Ccn2, Mest, Klf11, Xkr8, Meaf6, Mybphl, E130201H02Rik, Pithd1, Fbxo46, Jakmip1, Rpl39, Mfsd4b4, Klhl32, Pcyt2, Pdzd11	Pik3r6, Tmem267, Clec2g, P2ry14, Adam33, Arap1, Slc33a1, Klhl32, Hoxa5, Naa30, Wtip, Dapk1, Gm336, Grb10, Mfsd13a, Gins3, 0610009L18Rik, Fermt3, Gjd3, Napsa, Chst7, Anks6, Zfp280d, Prpf39, Katnbl1, 2900026A02Rik, Dtx1, Pced1b, BC048507, Acadl, Sox3, Nrbf2, Mest, Hgh1, Nrbf2, Sgsm3, 1700124L16Rik, B3gnt9, Cnksr2, Tasor, Magt1, Disc1, Mcm9, Zfp984, Foxred2, Kif28, Gm15554, 2310011J03Rik, Zfp872, Egfl7	Akap12, Mfap3, Tlr11, Gm18759, Vmn2r90, Rnase4, Blk, Soat2, Mir6924, Grb10, Olfr730, Prl3a1, Gm17966, Dtnbp1, Specc1l, H4c2, Gm9034, Rhoq, Slc27a6, Epcam, Ccar2, Trip10, Gm25102, Gipc3, Cenph, Fmnl3, Apobec3, Acox1, Gpr45, 4930578M01Rik, Myl4, Ankrd40, Gm17781, Nudt16l1, Lox, Diaph1, Esp3, Arl8a, Gh, Rpl31-ps17, Gm5532, Mad2l1bp, Dact1, Rab32, Traj23, Traj17, Pgap6, Pcbp3, Mir3961, 9130008F23Rik
Number of genes affected	3713	3863	6959

Adjusted p-value < 0.05 and abs (methylation difference) >=5%.

### 3.4 D1ManPrup3-SLIT treatment regulates methylation of genes linked to anaphylaxis suppression and immunomodulation

**Figure 3:**
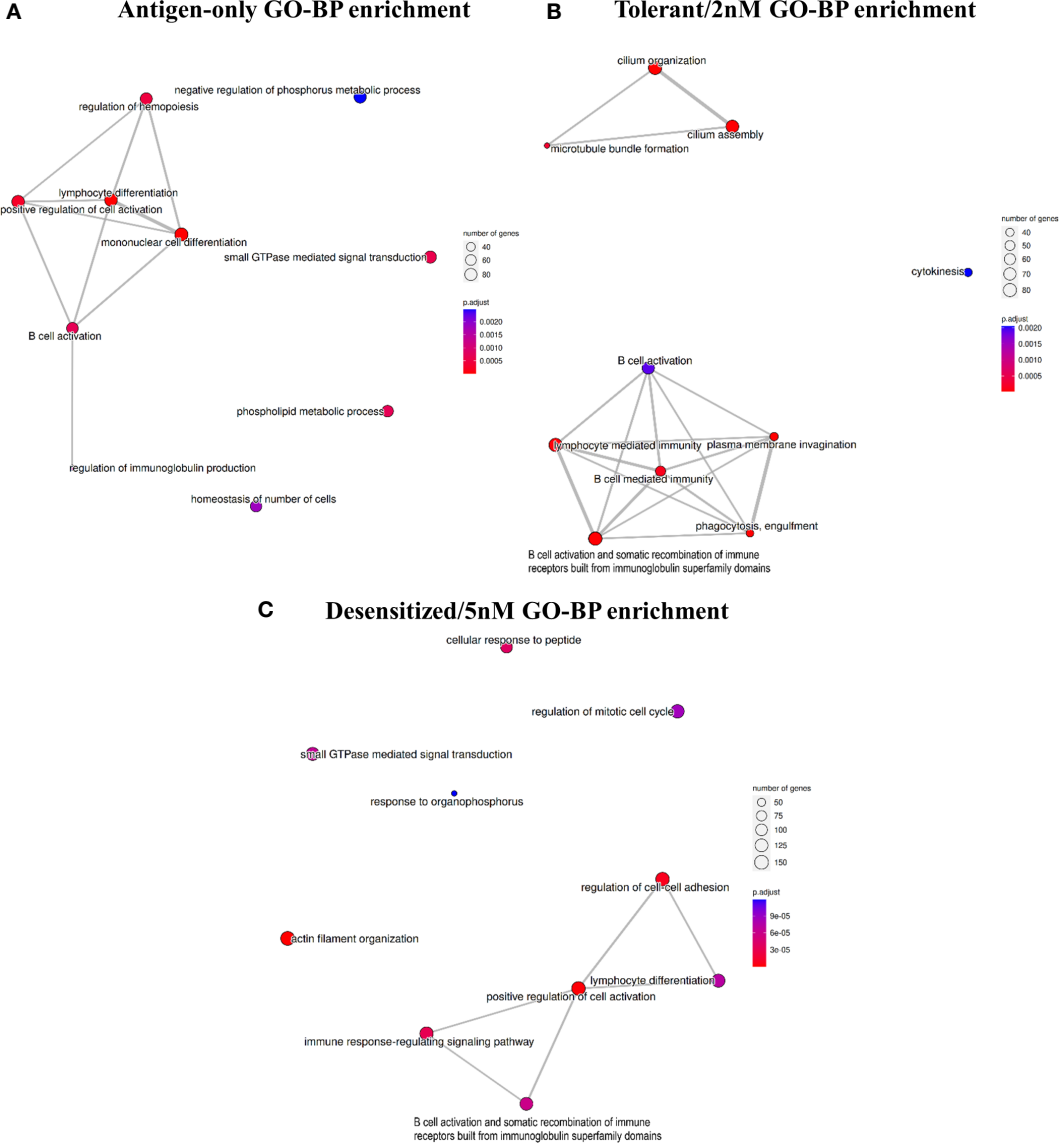
Top 10 enriched gene ontology terms in the biological processes category for each comparison: **(A)** Antigen-only versus Anaphylactic, **(B)** Tolerant/2nM versus Anaphylactic, and **(C)** Desensitized/5nM. Terms are connected if there were shared genes between the set of genes enriching each team.

Analyzing in detail the affected genes and pathways, **Figure 3** represents for each comparison the top 10 of enriched gene ontology (GO) biological processes terms and its clusterization based on the genes enriching each term. In the three comparisons, the top-tier functional enrichment presents a principal cluster of immune-related GO terms revealing that DMPRs detected could be linked to the absence of anaphylactic symptoms at challenge.

As expected, methylation profile of Antigen-only group differed from Anaphylactic mainly on genes involved on the immune function, as Antigen-only mice were never conditioned to develop anaphylaxis (**Figure 3A**). In the same line, D1ManPrup3-SLIT, and specifically D1ManPrup3 dosage, caused a divergence among methylation profiles of Tolerant, Desensitized, and Anaphylactic groups. Both groups showed DMPRs enriched terms linked to: i) DCs regulation of B cell mediated immunity and B cell effector mechanisms with terms such as B cell mediated immunity; ii) B cell activation and somatic recombination of immune receptors built from immunoglobulin superfamily domains (**Figure 3**). However, dosage also generated characteristic enrichment, showing in the comparison of Tolerant group top enriched terms included terms related to cilia and cytokinesis, which are part of DCs cellular behavior (**Figure 3B**). On the other hand, the comparison of Desensitized mice exhibited multiple terms apparently unrelated with each other and with unspecific involvement in cellular biology, such as regulation of mitotic cell cycle or small GTPase mediated signal transduction. Other terms enriched in Desensitized group such as cellular response to peptide and actin filament organization are more directly implicated with DCs involvement on antigen processing and presentation. Moreover, to study these GO deeply, **Table S2** shows all unique terms enriched for each D1ManPrup3-SLIT comparison. Among them, highlighting Tolerant/2nM the following: T-helper 1 cytokine production, regulation of miRNA transcription, regulation of hypersensitivity, regulation of leukocyte proliferation, myeloid leukocyte activation and phagocytosis recognition (**Table S2**). Whereas for Desensitized/5nM the terms that stand out are: interleukin-2 production, leukocyte cell-cell adhesion, regulation of toll-like receptor signaling pathway, and antigen processing and presentation of peptide antigen *via* MHC class II (**Table S2**).

### 3.5 The different SLIT dosage induces distinctive methylation changes related to immune-tolerance

**Figure 4:**
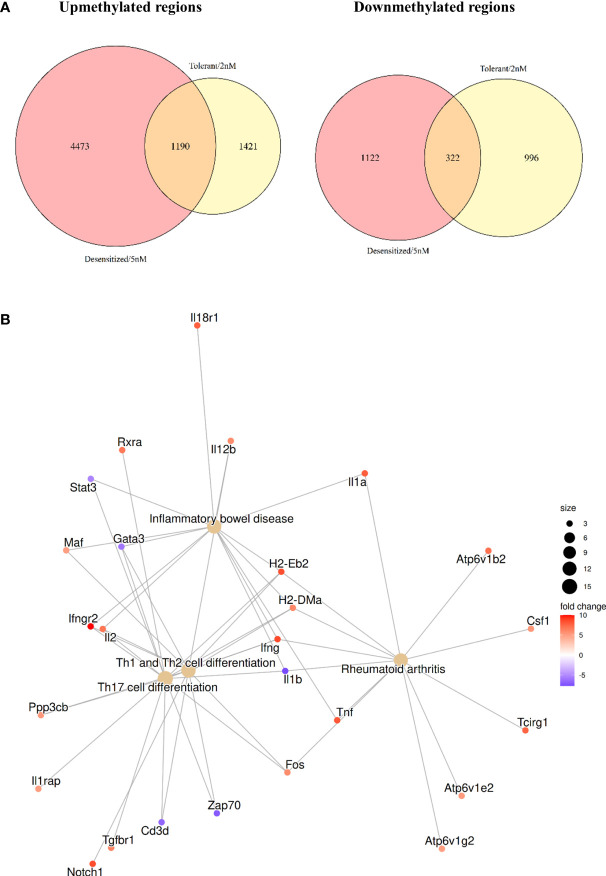
Comparison of the DMPRs found for each D1ManPrup3-SLIT group. **(A)** Venn diagrams contrasting upmethylated and downmethylated regions, respectively. **(B)** Highlighted enriched KEGG terms obtained with common D1ManPrup3-SLIT DMPRs.

Two Venn diagrams are shown in **Figure 4A**, comparing upmethylated and downmethylated DMPRs in Tolerant and Desensitized. Most of the DMPRs found were exclusive for each comparison, Desensitized group showed 1,122 downmethylated and 4,473 upmethylated regions, whereas Tolerant group exhibited 996 downmethylated and 1,421 upmethylated regions. On the contrary, the two D1ManPrup3-SLIT groups only shared 322 downmethylated and 1,190 upmethylated regions, compared to Anaphylactic group (**Figure 4A**). These results could corroborate the differences found in the functional enrichment described above (**Figure 3**). Exclusivity of genes affected with at least one DMPRs found in SLIT comparisons is showed in **Table S2**.

However, we have to take into account that at the time-point when the samples were collected, D1ManPrup3-SLIT mice, independently of the dose, tolerated Pru p 3 although this response diverge over the time towards tolerance or desensitization 5 weeks later. This indicates the possibility to find shared mechanisms of immune tolerance as well as some other different that will lead to either tolerance or desensitization at this early time point. To elucidate this phenomenon, a functional enrichment of the genes affected by the DMPRs present in both comparisons was performed. **Figure 4B** shows some highlighted KEGG enriched pathways to illustrate the epigenomic changes induced by the treatment that could be enhancing tolerance. The selected pathways are: Inflammatory bowel disease, Rheumatoid arthritis, Th17 cell differentiation, and Th1 and Th2 cell differentiation, which are enriched with a plethora of immune tolerance related genes such as cytokines (*Il1a*, *Il1b*, *Il2*, *Il12b*, *Ifng*, and *Tnf*) and proinflammatory transcription factors (*Fos*, *Maf*, and *Gata3*). In **Table S2** all enrichments for common core DMPRs are shown. Moreover, 466 of those genes (**Figure S2**) also had a DMPR in the Antigen-only group, which could further reinforce the involvement of those genes in the absence of anaphylaxis.

### 3.6 Differentially methylated regions affect to gene expression promoting Th1 differentiation and tolerance perpetuation

**Figure 5:**
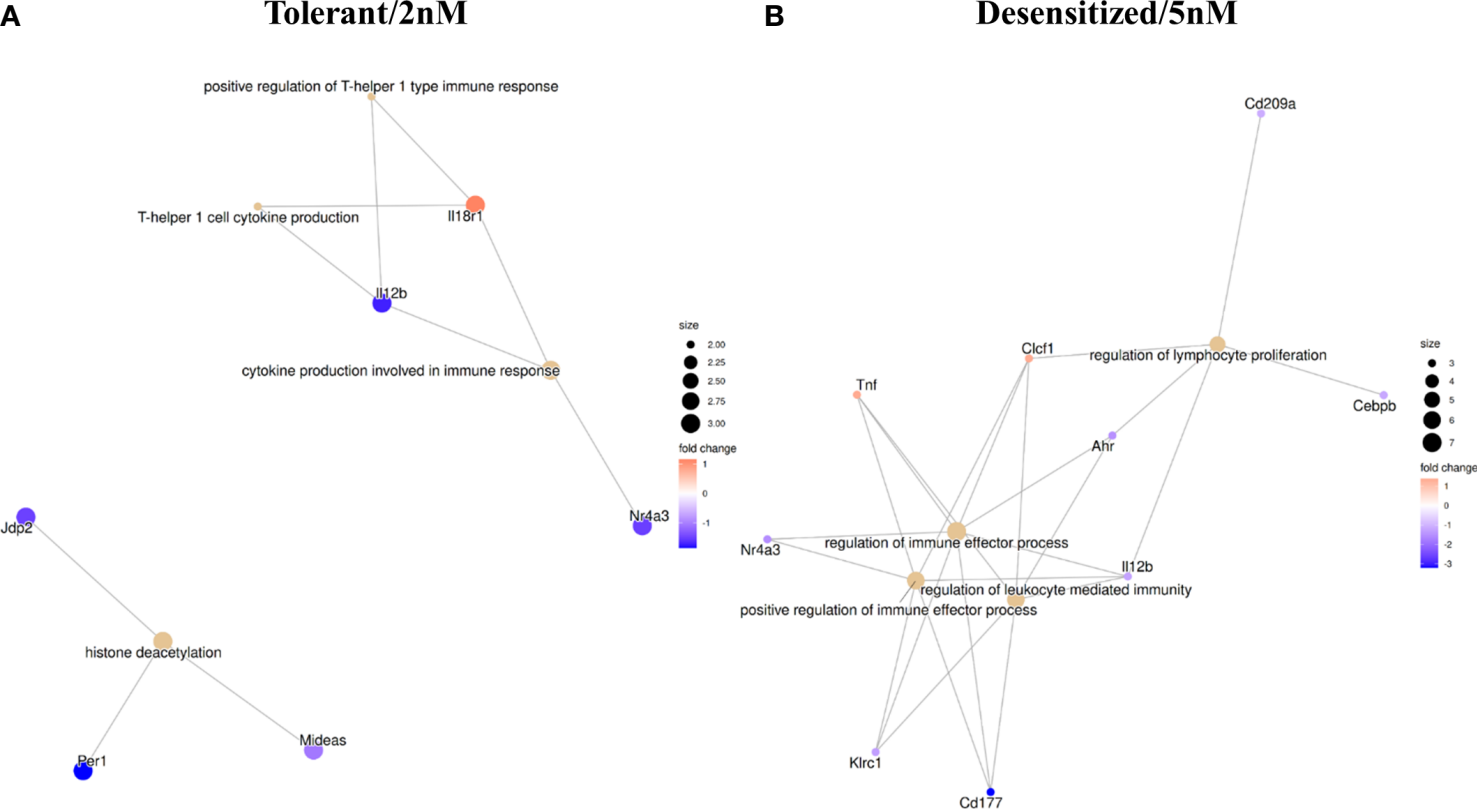
Functional enrichment in GO terms obtained with all differentially methylated promoter regions influencing genes found as differentially expressed in RNA-seq. **(A)** Tolerant/2nM vs Anaphylaxis comparison. **(B)** Desensitized/5nM vs Anaphylaxis comparison.

In a previous work, we demonstrated the gene expression changes that characterize Desensitized and Tolerant animals when they were compared to Anaphylactic group (13). Although it is difficult to correlate directly methylation with raw gene expression values from previous RNA-seq results, as data were obtained from different batches of mice, the DMPRs found in differential methylation analysis have been compared with the previously found DEGs; these analyses compared all groups to Anaphylactic group. **Figure 5** represents a network of the genes found as expressed and differentially methylated, which produce a significant functional enrichment. In Tolerant mice, 29 DEGs displayed at least one DMPR (**Table S3**), from which *Il12b* and *Il18r1* are noteworthy because they are related to Th1 response as seen in the GO terms enrichment (**Figure 5A**). Besides, three genes (*Jdp2*, *Per1* and *Mideas*) linked to histone deacetylation were found. On the other hand, 39 DEGs with at least one DMPR in Desensitized group were observed (**Table S3**), from which *Tnf*, *Il12b*, *Cebpb*, *Cd209a* and *Ahr* stand out as they are related to regulation of immune effector processes and lymphocyte proliferation (**Figure 5B**). **Table S3** shows all DEGs with DMPRs.

## 4 Discussion

Cytosine methylation patterns are a key element for the regulation of gene expression. Therefore, in this study an insight into a successful AIT effect on gene expression at cytosine methylation level is provided. DCs from lymphatic nodes of the AIT murine model are expected to govern tolerance (9); thus, characterizing the methylation changes in DCs after AIT could explain some of the underlying mechanisms in long-lasting tolerance. In addition, dosage applied at AIT treatment could lead to temporary desensitization or long-term tolerance, which provides the opportunity to study the methylation changes that could discern these states at molecular level. Therefore, in this work we found that SLIT in mice with different doses of D1ManPrup3 produces different responses, tolerance or desensitization, reflected in the induction of different DNA methylation changes.

Remarkably, although the short-term phenotypes maintain the unique methylation landscapes found with each treatment, the largest number of DMPRs were found in desensitized mice, compared to Anaphylactic group. Therefore, while the maintenance of tolerance seems to be negatively correlated to the number of changes found after treatment, it might be associated with the regulation of different pathways and signaling routes, which is further supported by the relatively low number of shared DMPRs (466) and the unique functional enriched terms found in each SLIT comparison. However, focusing on D1ManPrup3-SLIT comparisons, 1,666 common DMPRs were found and could be related to common triggered mechanisms providing tolerance near the end of treatment. The common core of DMPRs found in our study affects genes involved in the regulation of Th1 skewed immune response such as *Il12b*, *Ifng* and *Ifngr2* (32–34). Moreover, it affects genes encoding proinflammatory cytokines like *Il1a*, *Il1b*, *Il2*, *Il18r1*, *Il24*, *Il33*, *Litaf*, and *Tnf* (35–42), MHC-II genes including *H2-DMa*, *H2-Ke6*, *H2-Eb2*, and *H2-Q5* (43), antigen transport component *Vps37b* (44) and proinflammatory transcription factors such as *Maf*, *Junb*, *Nr4a3*, and *Gata3* (45–48). Tolerance is characterized by the reduction of Th2 responses and deviation towards Th1 response, and by the generation of Treg cells (49). Thus, the methylation changes found after SLIT could be related to tolerance responses.

Moreover, most of the genes detected in this work were previously observed as differentially expressed (DE) in an RNA-seq study by our group (13). Specifically, *Il12b* was differentially downregulated in both D1ManPrup3-SLIT groups when compared to the Anaphylactic one, which could mean an imbalance towards Th2 responses characteristic of anaphylactic reactions (50). In this study one upmethylated region in the CpG context adjacent to its promoter for Tolerant/2nM, and two smaller and more distant upmethylated regions for Desensitized/5nM, besides suggesting a canonical relation between upmethylation and reduced expression (51); *Il12b* downregulation was more pronounced in Tolerant/2nM and could be due to a the different DMPRs detected. Furthermore, differentially downregulated *Nr4a3*, *Vps37b*, and *Litaf*, confirmed D1ManPrup3-SLIT modification of DCs maturation/activation after challenge. Moreover, *Il18r1* was underexpressed in the RNA-seq in the Tolerant/2nM group, and in the present study was upmethylated in both D1ManPrup3-SLIT, which may be due to differences in size, magnitude, and location of the DMPRs. This receptor has been linked to enhanced Th1 and ablated Th2 responses (52) and generally, it can be involved in both anti-inflammatory and pro-inflammatory responses (53). As a result, *Il18r1* could be considered a potential biomarker, as well as *Il12b*, to distinguish tolerant and desensitized mice.

Taking all these data into consideration, dose-related changes could be of greater interest because they could potentially differentiate long-term outcomes. Comparing the Desensitized and Anaphylactic groups, 5,596 unique DMPRs were found, from which several upmethylated DMPRs could influence genes linked to DC activation. Among them, we found toll-like receptors (*Tlr4*, *Tlr3*, *Tlr8*, *Tlr5*), and other genes linked to its function including *Cd180* and *Tirap* (54); genes involved in IL-2 production from DC like *Cd247*, *Irf4*, and *Pde4b* (55); and other cytokines such as Th1 promoting *Il27* (56), Th2 enhancing *Il7* (57), and pro-cytotoxic *Il15* (58, 59). Assuming a canonical relation between CpG methylation and expression, these changes would result in a reduction of DC proinflammatory activity. Similarly, but with the opposite effect, *Il10ra* and *Pten* upmethylated DMPRs could be linked to a less tolerogenic behavior of the Desensitized/5nM DCs, either reducing IL-10 signaling (60) or mTOR-managed tolerance induction (61, 62), respectively. Additionally, two more upmethylated DMPRs also stood out, as they appeared as DE and downregulated in the RNA-seq study (13), which were the high-mannose glycoprotein receptor (*Cd209a*) and the proinflammatory transcription factor (*Cebpb*) (63). The first one codes for DC-SIGN, which modulate TLRs activity and, therefore, DCs antigen recognition (64, 65). Finally, two DMPRs, one upmethylated and one downmethylated, were detected in *Tnf* gene promoter. The downmethylated region is closer to the transcription start site and is probably the cause for *Tnf* gene overexpression in the RNA-seq. Pro-inflammatory *Tnf* overexpression after Pru p 3 challenge could be a side effect of the D1ManPrup3-SLIT modulation wearing out over time in desensitized animals.

Besides that, in Tolerant/2nM mice, 2,417 unique DMPRs were found, which included multiple upmethylated regions in the promoters of genes such as Th2 driving cytokine *Il13* (66), MHC-II genes such as *H2-Oa*, *H2-Q1*, and *H2-T23* (43), Il18 accessory receptor (*Il18rap*) which mediates high affinity binding of Il18 to its receptor (67), *Il23a* which encodes, together with *Il12b*, the interleukin IL-23. Although IL-23 signaling can activate the same responses as IL-12 triggering Th17 responses (68, 69), it is acting primary on CD4^+^T memory cells (70), which could a play role in tolerance responses.

Moreover, an upmethylated region was found in a DMPR in the gene of the subunit alpha of the IgE receptor, which is part of the initiation of the allergic response, and has already been appointed as potential biomarker of AIT efficacy (71). Additionally other genes affected with upmethylated regions are directly related to DC maturation and proinflammatory activity such as the AP-1 transcription factor Jun, CD28 and CTL4 binding membrane protein *Cd86* and vesicular trafficking key component *Rab30* (48, 72). Conversely, two genes with upmethylated regions, *Tgfb1* and *Tgfb2*, linked to tolerogenic behavior of DCs were found (73), assuming a canonical relation between methylation and expression, indicating less signaling promoting tolerance, although its expression has not been tested and methylation could be actually enhancing its expression. Moreover, multiple downmethylated DMPRs were found for Tolerant/2nM animals, which could be linked to the absence of anaphylaxis, including *Il18*, *Il15*, AP-1 related transcriptions factors *Batf* and *Batf2* (74), and immunoglobulin A gene (*Igha*), which is involved in oral tolerance (75). Finally, two DMPRs, one upmethylated and one downmethylated, stood out because the genes affected by them were DE. *Ahsa2* gene DMPR was downmethylated and its mRNA upregulated, and encodes a *Hsp90* activating co-chaperone (76). In turn, *Hsp90* inhibition prevents antigen presentation and IL-12 secretion by DCs (77). *Maf* gene translates into a mTOR controlled AP-1 complex related transcription factor that controls lymphocytes tolerance promoting actions (78), and regulates TLR4 mediated response in DCs (79). Our results showed an upmethylated DMPR and as an upregulated DE gene.

We are aware of the limitations of the study, which imply further studies to corroborate these results. For example, although differences were observed at the methylation and gene expression levels, these changes need to be confirmed at the protein expression and, subsequently, studying the mechanism where these genes are involved. Long-term retained epigenetic modifications enhancing tolerance should be studied, however dendritic cells exposed to D1ManPrup3-SLIT are not expected to survive that long. Future studies in mice would approach other key cell types involved in tolerance with longer lifespan such as T regulatory cells, including memory T regulatory cells. Moreover, although it is a *in vivo* study, it has been performed in allergic mice, so we need to transfer to human cells in order to check if these genes are also found dysregulated in humans and if they could be used as biomarkers of tolerance. Finally, these results should be tested in other allergic and inflammatory diseases, to corroborate if they can be extrapolated and used as universal markers of tolerance.

In conclusion, we found that different D1ManPrup3-SLIT dosage induces diverse methylation changes that could be linked to long-term phenotype differences. Some of these dose-exclusive differentially methylated promoter regions found in the long-term protected mice against a Pru p 3 are related to tolerant Th1 response such as *Il12b*, *Il18r1*, *Il23a*, and *Il18*. Other DMPRs associated with desensitized phenotype could be a result of the protection wearing off over time or causing this loss. One remarkable example of a proinflammatory cytokine increasing its expressions in this group is *Tnf*, which is affected by two different DMPRs. Interestingly, these differential changes, which were detected at specific time point (one week after last dose of D1ManPrup3-SLIT) with a tolerant response, could be used as biomarkers to predict the future response to treatment, long-lasting tolerance, or desensitization. Therefore, these particular methylation marks affecting DC effector genes could be used as potential biomarkers to distinguish a successful AIT from a temporary desensitization.

## Data availability statement

The data presented in the study are deposited in the Bioproject repository, accession number PRJNA905809 (https://www.ncbi.nlm.nih.gov/bioproject/PRJNA905809).

## Ethics statement

The animal study was reviewed and approved by Animal Experimentation Ethics Committee of Andalusian Centre for Nanomedicine and Biotechnology (BIONAND, Malaga, Spain).

## Author contributions

Conceptualization: MT, JC and CM. Methodology: RN, MR, CL-M, MM-A, FP, JR-S, JR and JC. Formal analysis: RN, MR and JC. Data curation: RN, MR, FP, JC and CM. Original draft preparation: RN, CL-M, MM-A, JC and CM. Review and editing: MT, JC and CM. Supervision: JR, MT, JC and CM. Funding acquisition: MT, JR and CM. All authors have read and agreed to the published version of the manuscript. All authors contributed to the article and approved the submitted version.
