# Supplementary material for: Methylation changes induced by a glycodendropeptide immunotherapy and associated to tolerance in mice
Source: Front Immunol. 2022 Dec 14;13:1094172. doi: 10.3389/fimmu.2022.1094172 (PMC9832389; doi:10.3389/fimmu.2022.1094172)
Supplement: Supplementary file 1 [file DataSheet_1.docx]

Supplementary Material

# Supplementary Figures and Tables

## Supplementary Figures


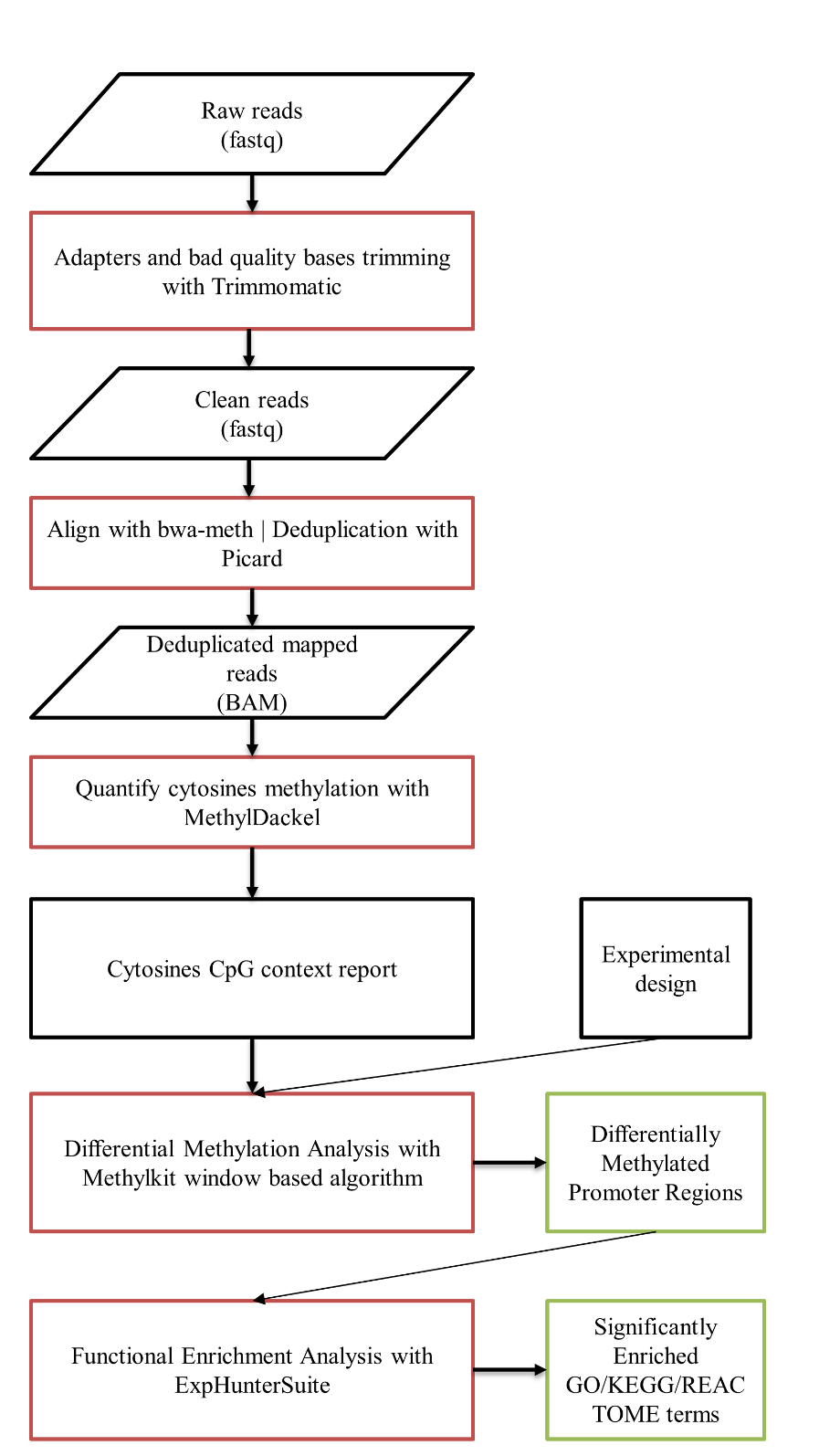


**Supplementary Figure 1.** Workflow applied in the methylation study.


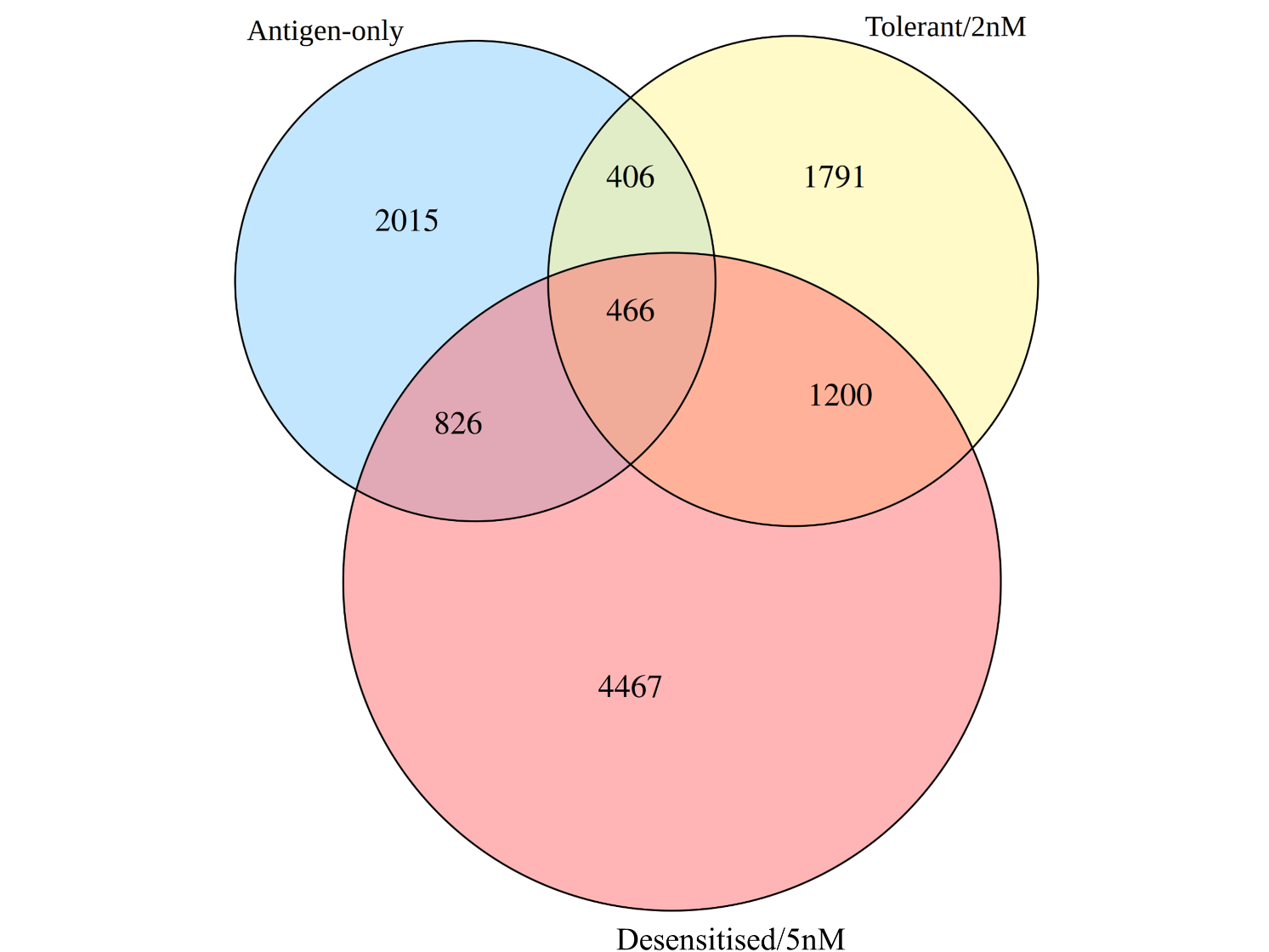


**Supplementary Figure 2.** Area proportional Venn Diagram of the DMPRs found in each comparison against Anaphylaxis group.
